# Blood flow kinetic energy is a novel marker for right ventricular global systolic function in patients with left ventricular assist device therapy

**DOI:** 10.3389/fcvm.2023.1093576

**Published:** 2023-05-16

**Authors:** Koichi Akiyama, Paolo C. Colombo, Eric J. Stöhr, Ruiping Ji, Isaac Y. Wu, Keiichi Itatani, Shohei Miyazaki, Teruyasu Nishino, Naotoshi Nakamura, Yasufumi Nakajima, Barry J McDonnell, Koji Takeda, Melana Yuzefpolskaya, Hiroo Takayama

**Affiliations:** ^1^ Department of Anesthesiology, Kindai University Hospital, Osakasayama, Japan; ^2^ Department of Medicine, Division of Cardiothoracic and Vascular Surgery, Columbia University Irving Medical Center, New York, NY, United States; ^3^ Department of Medicine, Division of Cardiology, Columbia University Irving Medical Center, New York, NY, United States; ^4^ COR-HELIX (CardiOvascular Regulation and Exercise Laboratory-Integration and Xploration), Institute of Sport Science, Leibniz University Hannover, Hannover, Germany; ^5^ Department of Anesthesiology, University of Rochester Medical Center, Rochester, NY, United States; ^6^ Department of Cardiovascular Surgery, Nagoya City University, Nagoya, Japan; ^7^ Cardio Flow Design, Tokyo, Japan; ^8^ iBLab (interdisciplinary Biology Laboratory), Division of Natural Science, Graduate School of Science, Nagoya University, Nagoya, Japan; ^9^ School of Sport & Health Sciences, Cardiff Metropolitan University, Cardiff, United Kingdom

**Keywords:** right ventricular failure, left ventricular assist device, vector flow mapping, kinetic energy, echocardiography

## Abstract

**Objectives:**

Right ventricular (RV) failure remains a major concern in heart failure (HF) patients undergoing left ventricular assist device (LVAD) implantation. We aimed to measure the kinetic energy of blood in the RV outflow tract (KE-RVOT) – a new marker of RV global systolic function. We also aimed to assess the relationship of KE-RVOT to other echocardiographic parameters in all subjects and assess the relationship of KE-RVOT to hemodynamic parameters of RV performance in HF patients.

**Methods:**

Fifty-one subjects were prospectively enrolled into 4 groups (healthy controls, NYHA Class II, NYHA Class IV, LVAD patients) as follows: 11 healthy controls, 32 HF patients (8 NYHA Class II and 24 Class IV), and 8 patients with preexisting LVADs. The 24 Class IV HF patients included 21 pre-LVAD and 3 pre-transplant patients. Echocardiographic parameters of RV function (TAPSE, St', Et', IVA, MPI) and RV outflow color-Doppler images were recorded in all patients. Invasive hemodynamic parameters of RV function were collected in all Class IV HF patients. KE-RVOT was derived from color-Doppler imaging using a vector flow mapping proprietary software. Kruskal-Wallis test was performed for comparison of KE-RVOT in each group. Correlation between KE-RVOT and echocardiographic/hemodynamic parameters was assessed by linear regression analysis. Receiver operating characteristic curves for the ability of KE-RVOT to predict early phase RV failure were generated.

**Results:**

KE-RVOT (median ± IQR) was higher in healthy controls (55.10 [39.70 to 76.43] mW/m) than in the Class II HF group (22.23 [15.41 to 35.58] mW/m, *p *< 0.005). KE-RVOT was further reduced in the Class IV HF group (9.02 [5.33 to 11.94] mW/m, *p *< 0.05). KE-RVOT was lower in the LVAD group (25.03 [9.88 to 38.98] mW/m) than the healthy controls group (*p *< 0.005). KE-RVOT had significant correlation with all echocardiographic parameters and no correlation with invasive hemodynamic parameters. RV failure occurred in 12 patients who underwent LVAD implantation in the Class IV HF group (1 patient was not eligible due to death immediately after the LVAD implantation). KE-RVOT cut-off value for prediction of RV failure was 9.15 mW/m (sensitivity: 0.67, specificity: 0.75, AUC: 0.66).

**Conclusions:**

KE-RVOT, a novel noninvasive measure of RV function, strongly correlates with well-established echocardiographic markers of RV performance. KE-RVOT is the energy generated by RV wall contraction. Therefore, KE-RVOT may reflect global RV function. The utility of KE-RVOT in prediction of RV failure post LVAD implantation requires further study.

## Introduction

The increasing number of patients with advanced heart failure has resulted in longer waiting times and increased mortality for patients listed for heart transplantation (1, 2). Due to the limited number of available organs, left ventricular assist device (LVAD) implantation has been used as an effective alternative to heart transplantation (3, 4). LVAD therapy improves outcomes in patients with advanced heart failure, especially after the introduction of continuous-flow LVAD technology in 2008 (5, 6). Although LVAD support improves exercise tolerance and reduces end-organ dysfunction, right ventricular failure (RVF) post-LVAD implantation continues to be a major cause of poor post-operative outcomes. The incidence of RVF is reported to be between 10% and 40% and is associated with increased mortality, morbidity, and hospital length of stay (7–9). Additional RVAD support is required in a proportion of patients with post-LVAD RVF. However, emergent conversion of LVAD support to biventricular mechanical circulatory support results in worse outcomes compared to elective establishment of biventricular mechanical circulatory support (10, 11). As a result, various models that utilize hemodynamic and echocardiographic parameters to predict post-LVAD RVF preoperatively, have been proposed (7, 12–24). No single prediction tool has gained universal support.

The kinetic energy of blood in the RV outflow tract (KE-RVOT)—a new marker of RV global systolic function—is a dynamic pressure that reflects the energy generated by the entire RV. We aimed to assess the relationship between KE-RVOT and well-established echocardiographic and hemodynamic parameters of RV performance. We also aimed to investigate whether KE-RVOT predicts RV failure post-LVAD implantation.

## Methods

### Patient population

This prospective study was approved by the institutional review board of our institution, and written informed consent was obtained from all participants. Healthy volunteers, outpatients with heart failure (NYHA Class II) or with an LVAD already implanted, and inpatients with heart failure (NYHA Class IV) were enrolled between November 2017 and March 2019.

### Echocardiographic and hemodynamic parameters

Echocardiographic parameters of RV function - tricuspid annular plane systolic excursion (TAPSE), St', Et', isovolumic acceleration (IVA), myocardial performance index (MPI)) - were assessed in accordance with published guidelines (25). Parasternal RV outflow views with color Doppler were recorded using transthoracic echocardiography (TTE) on all subjects (25). MPI was assessed using the tissue Doppler method, not the pulsed wave Doppler method. KE-RVOT was derived from the color Doppler parasternal RV outflow image using iTECHO® (Cardio Flow Design, Tokyo, Japan), a vector flow mapping (VFM) software. Invasive hemodynamic parameters of RV function - central venous pressure (CVP), pulmonary capillary wedge pressure (PCWP), systolic pulmonary artery pressure (sysPAP), diastolic pulmonary artery pressure (diaPAP), mean pulmonary artery pressure (mPAP), RV stroke volume - were collected during right heart catheterization in the patients with NYHA Class IV heart failure. RV stroke work index (RVSWi) and pulmonary artery pulsatility index (PAPi) were calculated from invasive hemodynamic parameters (16, 26). RVSWi was calculated as: [(mPAP – CVP)  ×  RV stroke volume index  ×  0.0136] mmHg・liter/m^2^. PAPi was calculated as: [(sysPAP– diaPAP)/CVP]. RV failure risk score (RVFRS) was also assessed in the patients with NYHA Class IV heart failure (12). Among the subjects with NYHA Class IV HF, those who underwent LVAD implantation were followed and assessed for RVF. Post-LVAD RVF was defined as the need for intravenous inotropic support for >14 days, inhaled nitric oxide for ≧48 h, right-sided circulatory support (extracorporeal membrane oxygenation or right ventricular assist device), or hospital discharge with an intravenous inotropic medication. The decision to utilize these interventions was made by the treating physician and was based on clinical signs of RV dysfunction.

### Image acquisition and determination of KE-RVOT

**Figure 1:**
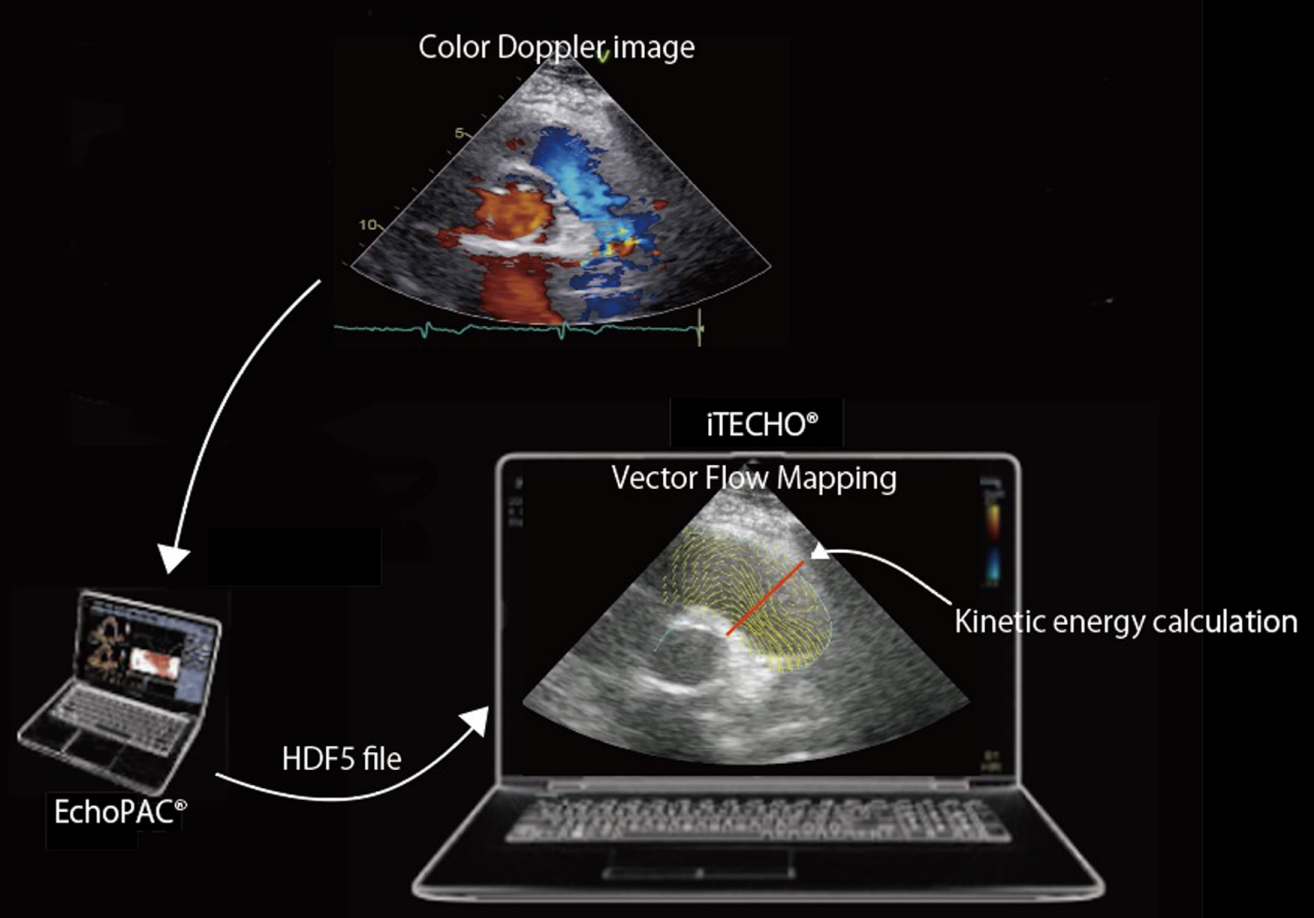
Color Doppler cine-loop image of the parasternal RV outflow view and its corresponding Vector Flow Mapping image. The stored cine-loop image is transferred to EchoPAC® (GE Healthcare, Chicago, USA) and converted into HDF-5 files. The HDF-5 file is imported into the VFM software (iTECHO®) and analyzed. Kinetic energy can be calculated from the vectors passing through RVOT (red line) over one cardiac cycle.

Echocardiographic parameters were assessed, and color Doppler images were stored using a standard diagnostic ultrasound system, Vivid E95 (GE Healthcare, Chicago, USA). To calculate KE-RVOT, color Doppler images were processed using the VFM software. Digitized two-dimensional color Doppler cine-loop images were obtained in the parasternal RV outflow view. Images were stored with the VFM configuration, the region of interest was maximized, and the Nyquist limit was set to mitigate aliasing. The ultrasound frequency was 3 MHz, with a frame rate of 30–40 using an M5Sc-D probe. The stored cine-loop images were transferred to EchoPAC® (GE Healthcare, Chicago, USA) and converted into HDF-5 files. The HDF-5 files were imported into the VFM software and analyzed. One cardiac cycle was selected for analysis by using two consecutive QRS complexes from the electrocardiogram as the beginning and end points. The right ventricular cavity-endocardial border and pulmonary artery wall were manually traced on the initial frame, and two-dimensional wall tracking was applied to detect wall motion (Figure 1). If the aliasing phenomenon was observed in the cine-loop images, the aliased pixels were manually corrected. Kinetic energy values were calculated from the vectors passing through RVOT over one cardiac cycle and averaged over three cardiac cycles.

### Principles of vector flow mapping

Velocity vectors of intraventricular blood flow are visualized by a two-dimensional continuity equation applied to color Doppler echocardiography of blood flow and wall-tracking method of the myocardium boundary, optical flow method (27–31). The velocity vectors of each pixel that are calculated from both the left-side and right-side boundaries are integrated by summation of the vectors according to a weight function (28). The KE-RVOT can be calculated according to the following equation:

$$KE=\int \frac{1}{2}\rho v^{2}\;\times \;vdL,$$

where *ρ* is the density of the blood (1,060 kg/m^3^), *v* is the velocity vector of the blood flow, and *dL* is an minute increment of the cross-sectional line (29).

### Statistical analysis

Statistical analysis was performed using JMP software (version 12.0.1 for Macintosh, from SAS). Continuous variables are represented as the median ± IQR. Kruskal-Wallis test was performed for comparison of each group. Tukey Kramer test was performed for further analysis if significant difference was confirmed. Correlation between KE-RVOT and echocardiographic/hemodynamic parameters was assessed by linear regression analysis. Receiver operating characteristic curves for the ability of the KE-RVOT, CVP/PCWP, RVSWi, PAPi, and RVFRS to predict early post-LVAD RVF were generated. *p* values <0.05 were considered to indicate significant differences.

## Results

### Patient characteristics

51 subjects were prospectively enrolled and separated into 4 groups: 11 in the healthy control group (C), 8 in the NYHA Class II group (II), 24 in the NYHA Class IV group (IV), and 8 with preexisting LVADs (LVAD). Among the 24 subjects in group IV, 21 subjects subsequently underwent LVAD implantation, and 3 subjects subsequently underwent orthotopic heart transplantation. Patients' clinical characteristics are shown in Table 1. There were no significant differences in the baseline characteristics between the different groups, except for LVEDD, LVESD, LVEF. There were 10 INTERMACS 2 subjects and 14 INTERMACS 3 subjects in group IV (Table 2). In group IV, all patients who subsequently underwent LVAD implantation received a HM3 device. In the group with preexisting LVADs, 6 subjects had a HM II device and 2 subjects had a HM3 device (Table 2).

**Table 1:** Patients' clinical characteristics.

	Healthy (*n* = 11)	NYHA II (*n* = 8)	NYHA IV (*n* = 24)	with LVAD (*n* = 8)	*p*-value
Age	52.8 ± 9.2	58.1 ± 11.4	59.5 ± 12.9	60.1 ± 18.0	0.31
Gender (male)	9 (81.8%)	4 (50%)	4 (83.3%)	6 (75%)	0.27
Height (cm)	169.6 ± 5.1	168.9 ± 5.2	174.8 ± 6.6	170.1 ± 10.4	0.05
Weight (kg)	70.6 ± 8.7	93 ± 39.1	88.7 ± 19.1	92.6 ± 40.7	0.16
BMI (kg/m^2^)	24.5 ± 2.1	32.3 ± 11.6	29.0 ± 5.1	31.1 ± 10.5	0.08
BSA (m^2^)	1.81 ± 0.13	2.00 ± 0.39	2.04 ± 0.23	2.02 ± 0.45	0.14
LVEDD (mm)	41.7 ± 5.4^a^	59.0 ± 9.1	63.6 ± 9.9	57.1 ± 9.4	<0.0001
LVESD (mm)	26.6 ± 3.7^a^	49.8 ± 8.9	56.0 ± 9.4	50.9 ± 11.9	<0.0001
LVEF (mm)	64.2 ± 3.5^a^	29.5 ± 8.1	19.3 ± 6.2	20.5 ± 5.3	<0.0001
Systolic BP (mmHg)	123 ± 11	117 ± 23	111 ± 15	111 ± 20	0.15
Diastolic BP (mmHg)	78 ± 8	71 ± 12	72 ± 12	82 ± 8	0.05
Etiology					0.96
Ischemic		2	7	2	
Nonischemic		6	17	6	

^a^ LVEDD, LVESD, and LVEF were significantly different in the healthy controls group compared to other groups.

**Table 2:** INTERMACS profile and LVAD device in the NYHA Class IV group and preexisting LVAD group.

	NYHA IV (*n* = 24)	With LVAD (*n* = 8)
INTERMACS 1	0	N/A
INTERMACS 2	10	N/A
INTERMACS 3	14	N/A
HM II/HM3	0 / 21	6 / 2

### Kinetic energy of the RVOT

**Figure 2:**
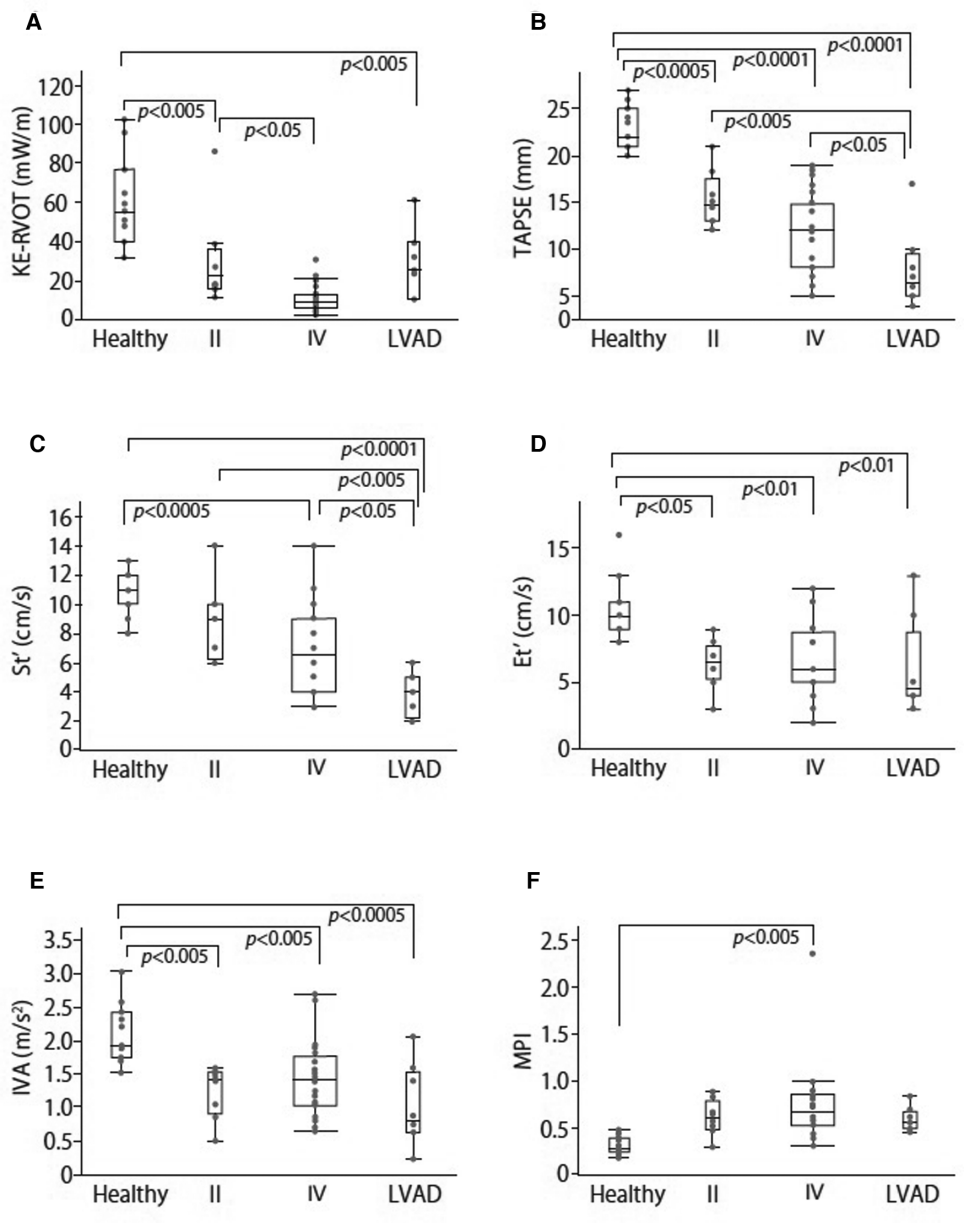
Box-and whisker plot compares KE-RVOT (**A**), TAPSE (**B**), St' (**C**), Et' (**D**), IVA (**E**), and MPI (**F**) values between each group.

KE-RVOT was significantly higher in group C (55.10 [39.70 to 76.43] mW/m) than in group II (22.23 [15.41 to 35.58] mW/m, *p *< 0.005), group IV (9.02 [5.33 to 11.94] mW/m, *p *< 0.0001), and the preexisting LVAD group (25.03 [9.88 to 38.98] mW/m, *p *< 0.005) (Figure 2A). KE-RVOT in group IV was also significantly lower than in group II (*p *< 0.05) (Figure 2A).

### Echocardiographic parameters

The TAPSE values of were 22 [21 to 25], 14.8 [13 to 17.53], 12 [8.1 to 14.75], and 6.5 [5 to 9.5] in groups C, II, IV, and LVAD respectively. There were significant differences between groups C and II (*p *< 0.0005), groups C and IV (*p *< 0.0001), groups C and LVAD (*p *< 0.0001), groups II and LVAD (*p *< 0.005), and groups IV and LVAD (*p *< 0.05) (Figure 2B). The values of St' were 11 [10 to 12], 9 [6.25 to 10], 6.5 [4 to 9], and 4 [2.25 to 5] in groups C, II, IV, and LVAD respectively. There were significant differences between groups C and IV (*p *< 0.0005), groups C and LVAD (*p *< 0.0001), groups II and LVAD (*p *< 0.005), and groups IV and LVAD (*p *< 0.05) (Figure 2C). The values of Et' were 10 [9 to 11], 6.5 [5.25 to 7.75], 6 [5 to 8.75], and 4.5 [4 to 8.75], in groups C, II, IV, and LVAD respectively. There were significant differences between in groups C and II (*p *< 0.05), groups C and IV (*p *< 0.01), and groups C and LVAD (*p *< 0.01) (Figure 2D). The values of IVA were 1.93 [1.73 to 2.42], 1.42 [0.9 to 1.53], 1.42 [1.02 to 1.77], and 0.81 [0.62 to 1.53] in groups C, II, IV, and LVAD respectively. There were significant differences between groups C and II (*p *< 0.005), groups C and IV (*p *< 0.005), and groups C and LVAD (*p *< 0.0005) (Figure 2E). The values of MPI were 0.28 [0.24 to 0.39], 0.6 [0.48 to 0.78], 0.67 [0.52 to 0.86], and 0.56 [0.49 to 0.67] in groups C, II, IV, and LVAD respectively. There was a significant difference between groups C and IV (*p *< 0.005) (Figure 2F).

### Hemodynamic parameters

In the patients with NYHA Class IV HF, CVP/PCWP, RVSWi, and PAPi were 0.39 [0.33 to 0.52], 6.68 [5.23 to 7.79], and 3.32 [2.22 to 5.89] respectively. As for RVFRS, 17 patients had score 0, 1 patient had score 2, 2 patients had score 2.5, and 1 patient had score 4.5.

### Correlation of KE-RVOT with other parameters

KE-RVOT had significant correlation with all echocardiographic parameters and no correlation with invasive hemodynamic parameters (Table 3).

**Table 3:** Correlation of KE-RVOT with echocardiographic parameters and invasive hemodynamic parameters.

Parameters	Correlation coefficient	*p* value
TAPSE	0.59	<0.0001
St'	0.36	<0.01
Et’	0.37	<0.01
IVA	0.42	<0.005
MPI	−0.43	<0.005
CVP/PCWP	0.78	0.12
RVSWi	0.77	0.07
PAPi	0.38	0.18
RVFRS	−0.85	0.07

### RV failure prediction

**Figure 3:**
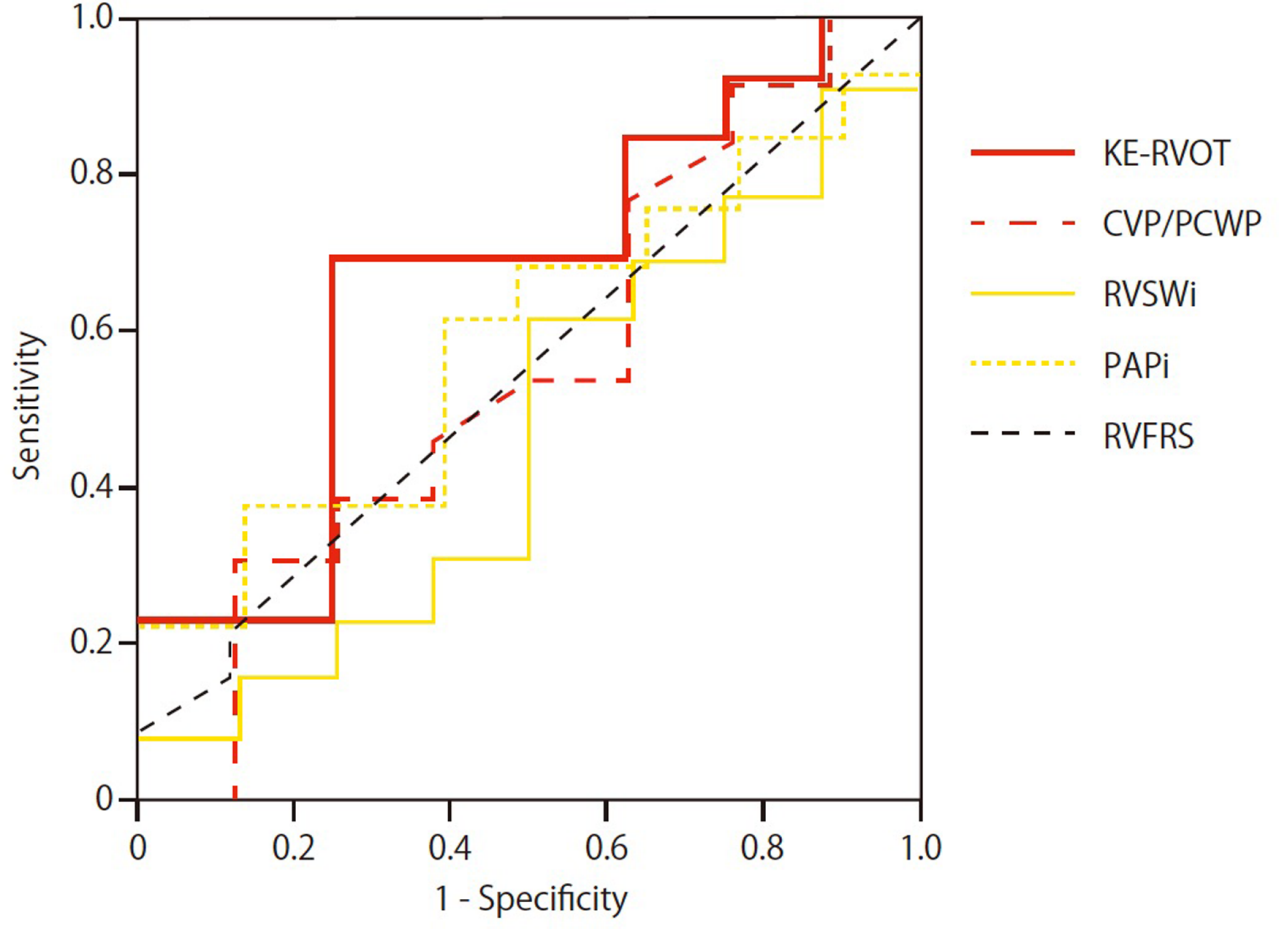
Receiver operating characteristic (ROC) curves and area under the curve (AUC) are shown for KE-RVOT, CVP/PCWP, RVSWi, PAPi, and RVFRS for prediction of RVF in the group IV patients undergoing LVAD implantation.

RV failure occurred in 12 patients among those who underwent LVAD implantation (1 patient was not eligible due to mortality immediately after the LVAD implantation). The overall performance for the prediction of RVF was greatest for KE-RVOT (AUC KE-ROVT 0.66; CVP/PCWP 0.56; RVSWi 0.47; PAPi 0.61; RVFRS 0.55) (Figure 3). Sensitivity and specificity were optimal with a KE-RVOT cut-off 9.15 mW/m (sensitivity: 0.69, specificity: 0.75).

## Discussion

Orthotopic heart transplantation is the most effective treatment for end-stage heart failure (32). Due to the limited number of donors, many potential recipients die before transplantation. LVAD support has been utilized as an alternative destination therapy for end-stage heart failure patients. However, RVF is a significant and frequent complication in the postoperative period after LVAD implantation, and its prediction is still difficult. We evaluated a novel parameter, KE-RVOT, using vector flow mapping, as a potential marker for RVF in heart failure patients.

This study demonstrates that KE-RVOT can be used as an indicator of RV function and may be useful as a predictor of post-LVAD RVF. KE-RVOT was significantly lower in the heart failure groups and the preexisting LVAD group compared with healthy controls. The significant reduction in KE-RVOT in the preexisting LVAD group compared to healthy controls may be due to a reduction in RV pulsatility in the preexisting LVAD group. Reduced pulsatility decreases the peak velocity of flow and reduces kinetic energy. There was a non-significant increase in KE-RVOT in the preexisting LVAD group compared to groups II and IV, which is likely due a higher cardiac output in patients with preexisting LVADs. KE-RVOT in group IV was significantly lower than in group II. The overall findings indicate that KE-RVOT may reflect RV function.

Although there was significant correlation between KE-RVOT and traditional echocardiographic parameters such as TAPSE, St', IVA, and MPI, KE-RVOT is distinct from traditional echocardiographic parameters. Traditional echocardiographic parameters typically assess regional function. The RV is an anatomically complex three-dimensional structure. It is triangular in shape in sagittal section and crescent-shaped in cross section. Furthermore, RV shape and function are influenced by the interventricular septum, which in turn is affected by ventricular loading conditions. Therefore, it is difficult to assess global RV function with traditional echocardiographic parameters.

In contrast, KE-RVOT assesses the flow energy that the entire RV ejects into the RVOT, which reflects both global RV function and pulmonary vascular resistance. KE-RVOT is the hydrodynamic pressure generated by the whole RV pushing blood against pulmonary vascular resistance. Han et al. evaluated KE-RVOT in patients with pulmonary arterial hypertension (WHO functional class I or II) and healthy subjects using 4D flow MRI (33). They demonstrated that patients with pulmonary arterial hypertension had lower KE- RVOT than healthy subjects. RV ejection fraction was lower in the patients with pulmonary arterial hypertension than in the healthy subjects. The lower KE-RVOT of the patients with pulmonary arterial hypertension was thought to be due to both hypokinetic RV wall motion and high pulmonary vascular resistance. Their study also indicated that KE-RVOT reflects RV-PA coupling. Fredriksson et al. investigated the difference in KE-RV between patients with mild ischemic heart disease and healthy controls using 4D flow MRI (34). Although there was no significant RV functional difference between the patients with high left ventricular end diastolic volume index and healthy subjects based on conventional MRI and echocardiographic indices, KE-RV was lower in the patients with high left ventricular end diastolic volume index compared to the patients with low left ventricular end diastolic volume index and heathy subjects. They concluded that subtle impairment of RV function can be detected by KE-RV. Finally, Rao et al. underlined the importance of KE-RVOT because KE forms a greater proportion of the total energy in the pulmonary circuit when compared to the systemic circuit (the pressure in the pulmonary artery is one-sixth of the pressure in the aorta, but the KE is similar in magnitude in both vessels) (35).

KE-RVOT may also be a good predictor of RVF post-LVAD implantation similar to other well-known predictors such as CVP/PCWP, RVSWi, PAPi, RVFRS. Right to left ventricular end-diastolic diameter ratio is another predictor of RVF before isolated LVAD implantation, however we did not acquire the specific images needed to accurately calculate this ratio in our study (36). Elevated CVP and laboratory abnormalities related with congestion, and reduced PAP are the preoperative parameters that are associated with increased risk of RV failure (7, 37–42). However, several studies have shown that preoperative elevation of CVP does not reliably predict risk for RVF (37–42). Although PAPi is well-known index for RV function, we found that PAPi did not correlate with KE-RVOT. This may be due to the fact that PAP and CVP (pressure parameter) may be more susceptible to change depending on patient's condition than KE-RVOT (fluid dynamic parameter). Additionally, KE-RVOT may be a better marker for RV-PA coupling. In other hemodynamic parameters (CVP/PCWP, RVSWi) as well, since they are calculated from pressure information and volume information, they may be susceptible to change depending on the situation. Therefore, these hemodynamic parameters did not correlate with KE-RVOT. Regarding RVFRS, because it is an index based on both laboratory data and vasopressor requirement, it is likely that there was no correlation with KE-RVOT. Notably, CVP/PCWP, RVSWi, and PAPi are combined indices which require invasive pulmonary artery catheter placement. In contrast, KE-RVOT analysis can be done with only echocardiographic imaging.

### Study limitations

The analysis of KE-RVOT requires adequate color Doppler imaging of the parasternal RV outflow view with accurate delineation of the RVOT. The sample size in this study was limited. However, we were able to obtain adequate imaging in all subjects in this study. Larger prospective studies are needed to assess the usefulness of KE-RVOT as a marker for RV function and predictor of post-LVAD RVF.

## Conclusion

KE-RVOT is a novel noninvasive measure of RV function that differentiate patients at various degree of heart failure patients, and may carry prognostic implication for patients undergoing LVAD implantation. KE-RVOT may reflect global RV function. However, additional studies are required to further evaluate the KE-RVOT and its clinical role.

## Data Availability

The raw data supporting the conclusions of this article will be made available by the authors, without undue reservation.

## References

[1] Banner NR Bonser RS Clark AL Clark S Cowburn PJ Gardner RS UK guidelines for referral and assessment of adults for heart transplantation. Heart. (2011) 97:1520–7. 10.1136/heartjnl-2011-300048 21856726

[2] Mehra MR Kobashigawa J Starling R Russell S Uber PA Parameshwar J Listing Criteria for Heart Transplantation: International Society for Heart and Lung Transplantation Guidelines for the Care of Cardiac Transplant Candidates-2006. J Hear Lung Transplant. (2006) 25:1024–42. 10.1016/j.healun.2006.06.008 16962464

[3] Trivedi JR Cheng A Singh R Williams ML Slaughter MS. Survival on the heart transplant waiting list: Impact of continuous flow left ventricular assist device as bridge to transplant. Ann Thorac Surg. (2014) 98:830–4. 10.1016/j.athoracsur.2014.05.019 25087934

[4] Miller LW Pagani FD Russell SD John R Boyle AJ Aaronson KD Use of a Continuous-Flow Device in Patients Awaiting Heart Transplantation. N Engl J Med. (2007) 357:885–96. 10.1056/NEJMoa067758 17761592

[5] Shah N Agarwal V Patel N Deshmukh A Chothani A Garg J National trends in utilization, mortality, complications, and cost of care after left ventricular assist device implantation from 2005 to 2011. Ann Thorac Surg. (2016) 101:1477–84. 10.1016/j.athoracsur.2015.09.013 26588867

[6] Mehra MR Uriel N Naka Y Cleveland JC Yuzefpolskaya M Salerno CT A fully magnetically levitated left ventricular assist device — final report. N Engl J Med. (2019) 380:1618–27. 10.1056/NEJMoa1900486 30883052

[7] Dang NC Topkara VK Mercando M Kay J Kruger KH Aboodi MS Right heart failure after left ventricular assist device implantation in patients with chronic congestive heart failure. J Hear Lung Transplant. (2006) 25:1–6. 10.1016/j.healun.2005.07.008 16399523

[8] Scherer M Sirat AS Moritz A Martens S. Extracorporeal membrane oxygenation as perioperative right ventricular support in patients with biventricular failure undergoing left ventricular assist device implantation. Eur J Cardio-thoracic Surg. (2011) 39:939–44. 10.1016/j.ejcts.2010.09.044 21071240

[9] Aissaoui N Morshuis M Schoenbrodt M Hakim MK Kizner L Börgermann J Temporary right ventricular mechanical circulatory support for the management of right ventricular failure in critically ill patients. J Thorac Cardiovasc Surg. (2013) 146:186–91. 10.1016/j.jtcvs.2013.01.044 23434450

[10] Copeland JG Smith RG Bose RK Tsau PH Nolan PE Slepian MJ. Risk factor analysis for bridge to transplantation with the CardioWest total artificial heart. Ann Thorac Surg. (2008) 85:1639–44. 10.1016/j.athoracsur.2008.01.052 18442554

[11] Fitzpatrick JR Frederick JR Hiesinger W Hsu VM McCormick RC Kozin ED Early planned institution of biventricular mechanical circulatory support results in improved outcomes compared with delayed conversion of a left ventricular assist device to a biventricular assist device. J Thorac Cardiovasc Surg. (2009) 137:971–7. 10.1016/j.jtcvs.2008.09.021 19327526 PMC3232461

[12] Matthews JC Koelling TM Pagani FD Aaronson KD. The right ventricular failure risk score. A pre-operative tool for assessing the risk of right ventricular failure in left ventricular assist device candidates. J Am Coll Cardiol. (2008) 51:2163–72. 10.1016/j.jacc.2008.03.009 18510965 PMC2842901

[13] Kormos RL Teuteberg JJ Pagani FD Russell SD John R Miller LW Right ventricular failure in patients with the HeartMate II continuous-flow left ventricular assist device: Incidence, risk factors, and effect on outcomes. J Thorac Cardiovasc Surg. (2010) 139:1316–24. 10.1016/j.jtcvs.2009.11.020 20132950

[14] Atluri P Goldstone AB Fairman AS Macarthur JW Shudo Y Cohen JE Predicting right ventricular failure in the modern, continuous flow left ventricular assist device era. Ann Thorac Surg. (2013) 96:857–64. 10.1016/j.athoracsur.2013.03.099 23791165 PMC4111251

[15] Drakos SG Janicki L Horne BD Kfoury AG Reid BB Clayson S Risk Factors Predictive of Right Ventricular Failure After Left Ventricular Assist Device Implantation. Am J Cardiol. (2010) 105:1030–5. 10.1016/j.amjcard.2009.11.026 20346326

[16] Fitzpatrick JR Frederick JR Hsu VM Kozin ED O'Hara ML Howell E Risk score derived from pre-operative data analysis predicts the need for biventricular mechanical circulatory support. J Hear Lung Transplant. (2008) 27:1286–92. 10.1016/j.healun.2008.09.006 PMC3235680 19059108

[17] Puwanant S Hamilton KK Klodell CT Hill JA Schofield RS Cleeton TS Tricuspid annular motion as a predictor of severe right ventricular failure after left ventricular assist device implantation. J Hear Lung Transplant. (2008) 27:1102–7. 10.1016/j.healun.2008.07.022 18926401

[18] Potapov E V Stepanenko A Dandel M Kukucka M Lehmkuhi HB Weng Y Tricuspid incompetence and geometry of the right ventricle as predictors of right ventricular function after implantation of a left ventricular assist device. J Hear Lung Transplant. (2008) 27:1275–81. 10.1016/j.healun.2008.08.012 19059106

[19] Kukucka M Stepanenko A Potapov E Krabatsch T Redlim M Mladenow A Right-to-left ventricular end-diastolic diameter ratio and prediction of right ventricular failure with continuous-flow left ventricular assist devices. J Hear Lung Transplant. (2011) 30:64–9. 10.1016/j.healun.2010.09.006 21036066

[20] Vivo RP Cordero-Reyes AM Qamar U Garikipati S Trevino AR Aldeiri M Increased right-to-left ventricle diameter ratio is a strong predictor of right ventricular failure after left ventricular assist device. J Hear Lung Transplant. (2013) 32:792–9. 10.1016/j.healun.2013.05.016 23856216

[21] Kato TS Farr M Schulze PC Maurer M Shahzad K Iwata S Usefulness of two-dimensional echocardiographic parameters of the left side of the heart to predict right ventricular failure after left ventricular assist device implantation. Am J Cardiol. (2012) 109:246–51. 10.1016/j.amjcard.2011.08.040 22088200

[22] Grant ADM Smedira NG Starling RC Marwick TH. Independent and incremental role of quantitative right ventricular evaluation for the prediction of right ventricular failure after left ventricular assist device implantation. J Am Coll Cardiol. (2012) 60:521–8. 10.1016/j.jacc.2012.02.073 22858287

[23] Cameli M Lisi M Righini FM Focardi M Lunghetti S Bernazzali S Speckle tracking echocardiography as a new technique to evaluate right ventricular function in patients with left ventricular assist device therapy. J Hear Lung Transplant. (2013) 32:424–30. 10.1016/j.healun.2012.12.010 23498163

[24] Dandel M Potapov E Krabatsch T Stepanenko A Löw A Vierecke J Load dependency of right ventricular performance is a major factor to be considered in decision making before ventricular assist device implantation. Circulation. (2013) 128:14–23. 10.1161/CIRCULATIONAHA.112.000335 24030398

[25] Lang RM Badano LP Victor MA Afilalo J Armstrong A Ernande L Recommendations for cardiac chamber quantification by echocardiography in adults: an update from the American society of echocardiography and the European association of cardiovascular imaging. J Am Soc Echocardiogr. (2015) 28:1–39.e14. 10.1016/j.echo.2014.10.003 25559473

[26] Kang G Ha R Banerjee D. Pulmonary artery pulsatility index predicts right ventricular failure after left ventricular assist device implantation. J Hear Lung Transplant. (2016) 35:67–73. 10.1016/j.healun.2015.06.009 26212656

[27] Garcia D Juan JC Tanné D Yotti R Cortina C Bertrand É Two-dimensional intraventricular flow mapping by digital processing conventional color-doppler echocardiography images. IEEE Trans Med Imaging. (2010) 29:1701–13. 10.1109/TMI.2010.2049656 20562044

[28] Itatani K Okada T Uejima T Tanaka T Ono M Miyaji K Intraventricular flow velocity vector visualization based on the continuity equation and measurements of vorticity and wall shear stress. Jpn J Appl Phys. (2013) 52:07HF16. 10.7567/JJAP.52.07HF16

[29] Akiyama K Maeda S Matsuyama T Kainuma A Ishii M Naito Y Vector flow mapping analysis of left ventricular energetic performance in healthy adult volunteers. BMC Cardiovasc Disord. (2017) 17:21. 10.1186/s12872-016-0444-7 28068909 PMC5223342

[30] Hayashi H Akiyama K Itatani K Scott D Sanchez J Ferrari G A novel in vivo assessment of fluid dynamics on aortic valve leaflet using epi-aortic echocardiogram. Echocardiography. (2020) 37:323–30. 10.1111/echo.14596 32003907 PMC8151665

[31] Hayashi H Itatani K Akiyama K Zhao Y Kurlansky P Deroo S Influence of aneurysmal aortic root geometry on mechanical stress to the aortic valve leaflet. Eur Heart J Cardiovasc Imaging. (2021) 22:986–94. 10.1093/ehjci/jeab006 33611382 PMC8370567

[32] Taylor DO Edwards LB Boucek MM Trulock EP Aurora P Christie J Registry of the international society for heart and lung transplantation: twenty-fourth official adult heart transplant report-2007. J Hear Lung Transplant. (2007) 26:769–81. 10.1016/j.healun.2007.06.004 17692781

[33] Han QJ Witschey WRT Fang-Yen CM Arkles JS Barker AJ Forfia PR Altered right ventricular kinetic energy work density and viscous energy dissipation in patients with pulmonary arterial hypertension: A pilot study using 4D flow MRI. PLoS One. (2015) 10:1–14. 10.1371/journal.pone.0138365 PMC4587751 26418553

[34] Fredriksson AG Svalbring E Eriksson J Dyverfeldt P Alehagen U Engvall J 4D flow MRI can detect subtle right ventricular dysfunction in primary left ventricular disease. J Magn Reson Imaging. (2016) 43:558–65. 10.1002/jmri.25015 26213253

[35] Rao PS Awa S Linde LM. Role of kinetic energy in pulmonary valvar pressure gradients. Circulation. (1973) 48:65–73. 10.1161/01.CIR.48.1.65 4781250

[36] Kukucka M Stepanenko A Potapov E Krabatsch T Redlin M Mladenow A Right-to-left ventricular end-diastolic diameter ratio and prediction of right ventricular failure with continuous-flow left ventricular assist devices. J Hear Lung Transplant. (2011) 30:64–9. 10.1016/j.healun.2010.09.006 21036066

[37] Kavarana MN Pessin-Minsley MS Urtecho J Catanese KA Flannery M Oz MC Right ventricular dysfunction and organ failure in left ventricular assist device recipients: A continuing problem. Ann Thorac Surg. (2002) 73:745–50. 10.1016/S0003-4975(01)03406-3 11899176

[38] Farrar DJ Hill JD Pennington DG McBride LR Holman WL Kormos RL Preoperative and postoperative comparison of patients with univentricular and biventricular support with the thoratec ventricular assist device as a bridge to cardiac transplantation. J Thorac Cardiovasc Surg. (1997) 113:202–9. 10.1016/S0022-5223(97)70416-1 9011691

[39] Fukamachi K McCarthy PM Smedira NG Vargo RL Starling RC Young JB. Preoperative risk factors for right ventricular failure after implantable left ventricular assist device insertion. Ann Thorac Surg. (1999) 68:2181–4. 10.1016/S0003-4975(99)00753-5 10616999

[40] Kormos RL Gasior TA Kawai A Pham SM Murali S Hattler BG Transplant candidate's clinical status rather than right ventricular function defines need for univentricular versus biventricular support. J Thorac Cardiovasc Surg. (1996) 111:773–83. 10.1016/S0022-5223(96)70337-9 8614137

[41] Morgan JA John R Lee BJ Oz MC Naka Y. Is severe right ventricular failure in left ventricular assist device recipients a risk factor for unsuccessful bridging to transplant and post-transplant mortality. Ann Thorac Surg. (2004) 77:859–63. 10.1016/j.athoracsur.2003.09.048 14992887

[42] Santambrogioa L Bianchia T Fuardoa M Gazzoli F Veronesi R Braschi A Right ventricular failure after left ventricular assist device insertion: Preoperative risk factors. Interact Cardiovasc Thorac Surg. (2006) 5:379–82. 10.1510/icvts.2006.128322 17670597

